# Prevalencia y características de la enfermedad pulmonar obstructiva crónica en no fumadores

**DOI:** 10.1016/j.aprim.2022.102550

**Published:** 2022-12-27

**Authors:** Gabriela Mamani Trujillo, Kevin Clemente Chávez, Zulema Mansilla Sánchez, Carlos Andrés Mugruza-Vassallo

**Affiliations:** Escuela Profesional de Medicina Humana, Universidad Privada San Juan Bautista, Lima, Perú

*Sr. Editor:*

Por medio de la presente nos es grato dirigirnos ante usted para hacerle llegar nuestros comentarios sobre el artículo de «Prevalencia y características de la enfermedad pulmonar obstructiva crónica en no fumadores».

Si bien la enfermedad obstructiva crónica (EPOC) es considerada una enfermedad con una alta morbimortalidad a nivel mundial en los últimos años, ha sido reemplazada por una enfermedad que posee una mayor tasa de morbimortalidad en los últimos dos años. Actualmente esta enfermedad sigue siendo un problema de salud mundial al igual que la EPOC. Coincidimos en que la EPOC no siempre tiene relación con el tabaquismo, ya que hay pacientes que nunca han fumado y desarrollaron la enfermedad, por lo que debemos sugerir una búsqueda intensa de los factores a los cuales se asocia la EPOC^1^.

Para poder realizar el estudio clínico a los pacientes, se les realizó espirometría con test de broncodilatación en fase estable de la enfermedad, pero investigamos en otro artículo realizado sobre «Prevalencia de déficit de alfa-1 antitripsina en pacientes con EPOC en Argentina. Estudio DAAT.AR», en el cual realizaron a los pacientes espirometría pre y postinhalación del broncodilatador hallando mejores resultados para diagnosticar EPOC y usando la clasificación GOLD 2017 para poder clasificar el grado de obstrucción^2^.

Debemos tener en cuenta que el artículo «Prevalencia y características de la enfermedad pulmonar obstructiva crónica en no fumadores» fue realizado en 512 pacientes mayores de 40 años de edad, a comparación del artículo «Prevalencia de déficit de alfa-1 antitripsina en pacientes con EPOC en Argentina. Estudio DAAT.AR» que fue realizado en 3.254 pacientes mayores de 40 años, cabe decir que la diferencia de pacientes estudiados es notoria, esto influye en los resultados obtenidos^1^, ^2^.

Si bien es cierto que el segundo artículo fue realizado en población argentina, con ello no podemos intentar generalizar ni estandarizar que la causa y/o diagnóstico de EPOC será igual para el resto de población sea americana o de otras zonas más alejadas, pero sí concordamos que en ciertos grupos etarios y étnicos frecuentemente la etiología en común es por el tabaquismo.

Teniendo en cuenta el estudio de Rotterdam, donde se usaron pacientes mayores de 45 años, si bien la diferencia de edad es de cinco años, esto es un factor realmente influyente en la realización del estudio. De acuerdo con el estudio de Rotterdam, la prevalencia para desarrollar EPOC es superior en pacientes varones fumadores teniendo un porcentaje mínimo en mujeres^3^.

Es cierto que EPOC tiene diferentes etiologías desencadenantes, se deben tener en consideración los distintos factores no modificables como la raza, sexo y género, pues está demostrado que el sexo masculino es más propenso a desarrollar EPOC.

Al momento de comparar los tres estudios tenemos claro que la edad y el género son factores fundamentales para determinar la prevalencia en EPOC^2^, ^4^. Sin embargo, dos de los tres estudios descritos fueron realizados en distintos países teniendo rangos de edad, métodos de diagnóstico para EPOC y factores predisponentes, ambos estudios nos arrojan lo que el estudio de Rotterdam nos menciona, ya que es un estudio de cohorte con incidencia global.

## Conflicto de intereses

Los autores declaran no tener ningún conflicto de intereses.
